# Mucormycosis in a Diabetic Patient: A Case Report From Georgia

**DOI:** 10.7759/cureus.69399

**Published:** 2024-09-14

**Authors:** Luka Katsitadze, Giorgi Javakhishvili, George Burkadze, Sofia Nemsadze, Vakhtang Shiukashvili, George Kandelaki, Tinatin Gabrichidze, Tamar Didbaridze, Lia Janashvili

**Affiliations:** 1 Microbiology, Tbilisi State Medical University, Tbilisi, GEO; 2 Microbiology, Ken Walker International University, Tbilisi, GEO; 3 Pathology, Tbilisi State Medical University, Tbilisi, GEO; 4 Infectious Diseases, New Hospitals, Tbilisi, GEO; 5 Maxillofacial Surgery, New Hospitals, Tbilisi, GEO; 6 Epidemiology and Biostatics, Tbilisi State Medical University, Tbilisi, GEO; 7 Clinical Microbiology, The First University Clinic of Tbilisi State Medical University, Tbilisi, GEO; 8 Infectious Diseases, International University of Tbilisi, Tbilisi, GEO

**Keywords:** diabetes, treatment, diagnosis, rhizopus, mold

## Abstract

Mucormycosis is a rapidly progressive fungal infection that typically affects immunocompromised patients and diabetics. These fungi are found in soil and vegetation and can be easily inhaled by humans in day-to-day life; however, infection is rare unless specific risk factors are present. This case report presents a typical case of a 39-year-old male with poorly controlled type 1 diabetes mellitus. Initially presenting with a toothache in the upper molars, the infection quickly progressed to sinusitis, necrosis, and multiorgan failure. Based on our experience, prompt recognition of mucormycosis in risk-group patients is essential for improving future outcomes.

## Introduction

Mucormycosis in humans is usually caused by multiple genera from the *Mucoromycotina *subphylum, formerly regarded as “zygomycetes.” These are saprotrophic organisms commonly found worldwide on decaying matter in soil and vegetation. They are large, rapidly growing hyphae with wide-angle branching and few septations. The genera most commonly isolated from infected humans are *Rhizopus*, *Mucor*, *Lichtheimia*, *Cunninghamella*, *Apophysomyces,* and *Saksenaea *[1]. Estimating the incidence and prevalence of mucormycosis is challenging due to the low sensitivity of diagnostic studies and the fact that it is a non-reportable disease in the majority of the world [2]. Overall, the incidence of mucormycosis is increasing [3,4]. One population-based study in France found prevalence to be 0.7 cases per million in 1997 and 1.2 cases per million in 2006 [5]. Another study in Spain documented the rise in prevalence from 1.2 cases per 100,000 admissions (1988-2006) to 3.3 cases per 100,000 admissions (2007-2015) [6]. A review of 929 cases of mucormycosis in the English literature since 1885 established diabetes mellitus to be the most common underlying risk factor (36%), followed by malignancy (17%) and transplantation (7%) [7].

Rhino-orbito-cerebral infection is the most common presentation and likely originates from the inhalation of spores into the paranasal sinuses [8]. Diabetes mellitus and diabetic ketoacidosis are the most frequently encountered risk factors [7]. Initial symptoms include fever, headache, sinus pain, and nasal discharge. Observation of the necrotic tissue in the nasal mucosa, palate, and overlying skin is the hallmark of the disease. Less commonly encountered presentations include pulmonary, gastrointestinal, cutaneous, and renal mucormycosis. Dissemination is more common with immunocompromise and reaches mortality of up to 96% [7].

Early detection and treatment initiation are vital for improved outcomes. Histopathology is the gold standard for diagnosis, however, in a review of 929 cases of mucormycosis, only 50% were found to be culture-positive [7]. Early employment of advanced diagnostic techniques may facilitate diagnosis and improve disease outcomes in the future. In one study using polymerase chain reaction (PCR) as a diagnostic tool, *Mucorales *PCR was found to be positive in 22 out of 27 tissue specimens with initial histologic evidence of mucormycosis. *Mucorales *PCR also allowed the identification of 12 culture-negative cases in this cohort [9]. In a different study, the Bruker library and BMU database were used together for matrix-assisted laser desorption/ionization coupled to time-of-flight mass spectrometry (MALDI-TOF MS). This helped identify 90 (81.1%) of the isolates at the species level and 111 (100%) at the genus level, with a much higher success rate [10]. We present a case of rhinocerebral mucormycosis that was confirmed using a combination of histopathologic cultures and MALDI-TOF MS.

## Case presentation

On October 14, 2023, a 39-year-old male presented to the emergency department complaining of severe right-sided headache and malaise. The condition began a week before with a toothache in the right upper second and third molars. He had visited an ENT for his initial symptoms, who prescribed ceftriaxone (1 g twice a day) and metronidazole (500 mg twice a day) after an initial head CT. The patient showed no improvement with this regimen and decided to admit himself to the emergency department four days later.

Past medical history is significant for a 10-year history of type 1 diabetes mellitus managed with a basal-bolus insulin regimen. The patient admitted poor compliance with his medication for the past six months. His admission glucose was 383 mg/dL. An arterial blood gas (ABG) test demonstrated normal pH; urine was negative for ketones.

Physical examination demonstrated facial asymmetry due to right-sided periorbital swelling. Maxillary and frontal sinuses were tender to palpation. Rhinoscopy demonstrated swollen nasal mucosa and significant deformity of the nasal septum that nearly obstructed the left nasal passage. Vital signs were within normal limits. Initial differential diagnosis included orbital cellulitis, sinusitis, and dental abscess. Relevant laboratory findings on admission are presented in Table 1.

**Table 1:** Laboratory values on admission. CRP: C-reactive protein; INR: international normalized ratio

Parameters	Patient lab values	Normal lab values
Red blood cells	4.4 million/mm3	4.3-5.9 million/mm3
White blood cells	13.5 × 10^9^/L	4.5 × 10^9^/L-11 × 10^9^/L
Platelets	233 × 10^9^/L	150-450 × 10^9^/L
INR	1.1	0.8-1.1
CRP	82 mg/dL	<10 mg/dL
Glucose	143 mg/dL	70-99 mg/dL
Procalcitonin	0.06 ng/ml	0.1 ng/ml

**Figure 1:**
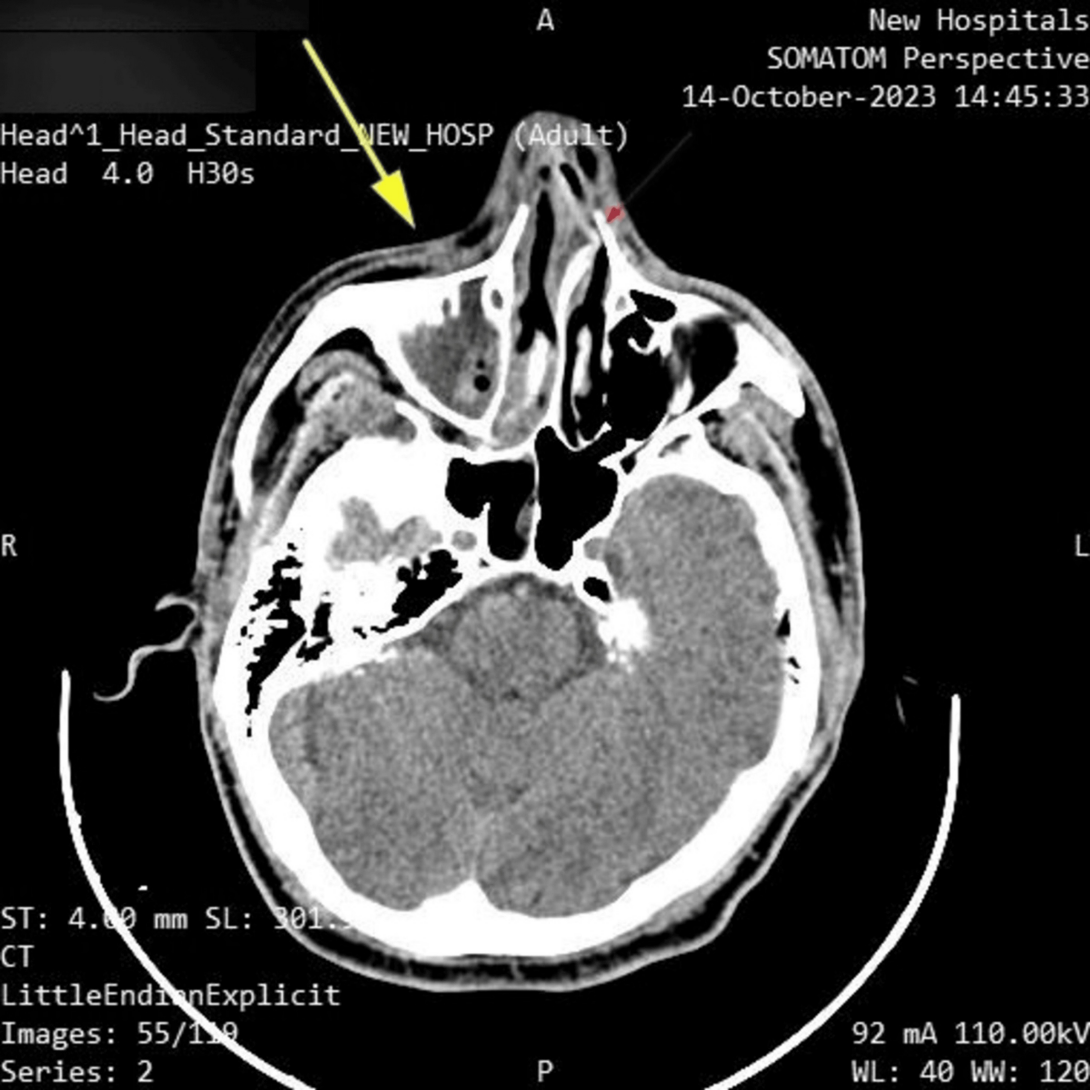
CT head axial Right-sided maxillary sinusitis (yellow arrow) and deformation of the nasal septum (red arrow).

**Figure 2:**
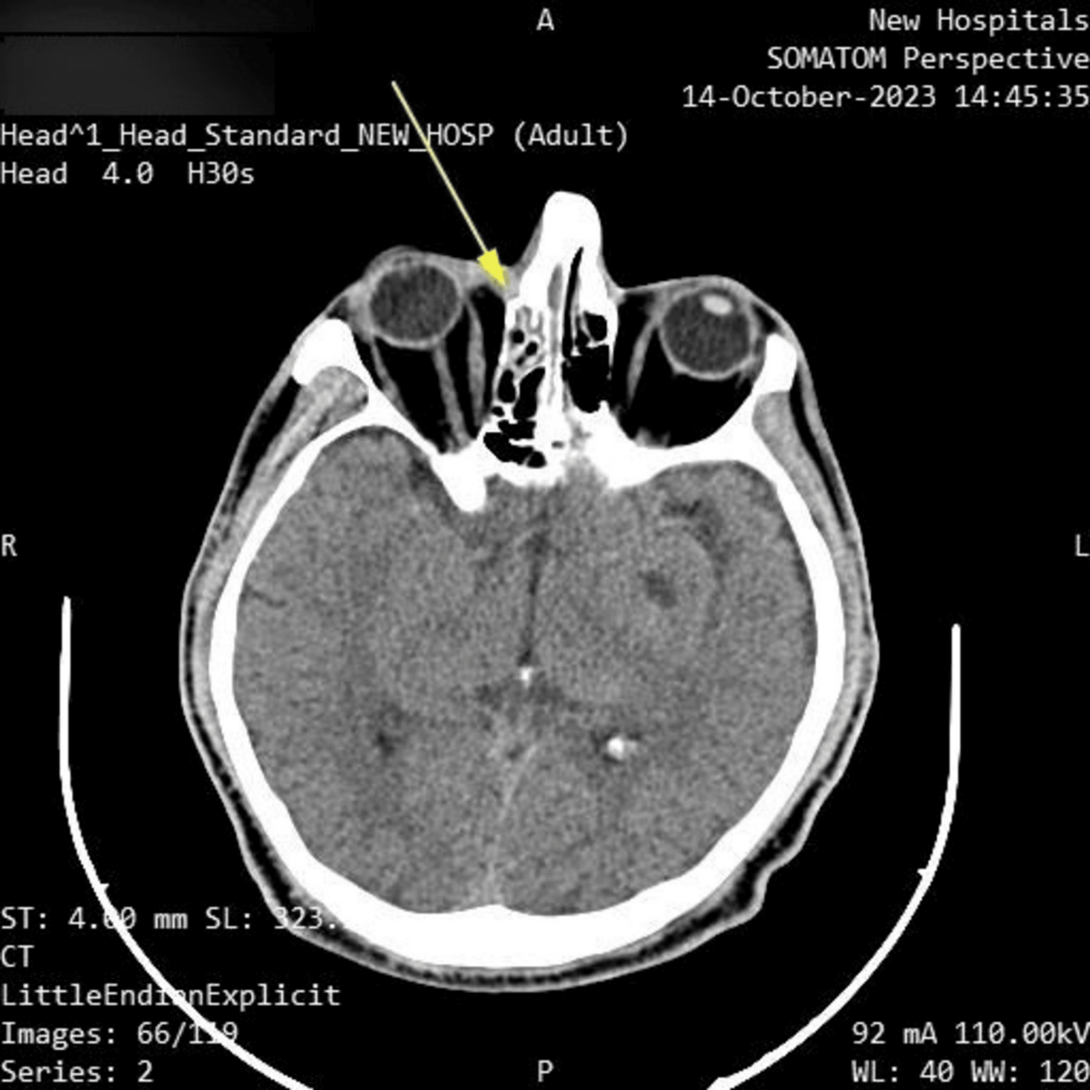
Axial CT head Right ethmoidal sinusitis (yellow arrow)

A CT scan without contrast was performed upon admission, revealing right-sided maxillary and ethmoidal sinusitis, along with swelling of the right paranasal and periorbital soft tissues. No changes were observed in the brain parenchyma. These findings are shown in Figures 1-2.

A maxillofacial surgeon and ENT were consulted. The oropharyngeal exam showed caries on the upper right molars and slight tenderness to percussion. Based on the patient's initial presentation of right-sided toothache and CT scan showing right-sided sinusitis, a decision was made to perform a radical antrostomy and extract affected teeth. Obtained samples were sent for bacteriological and histological analysis, and the patient was started on empiric antibiotic therapy with piperacillin-tazobactam.

**Figure 3:**
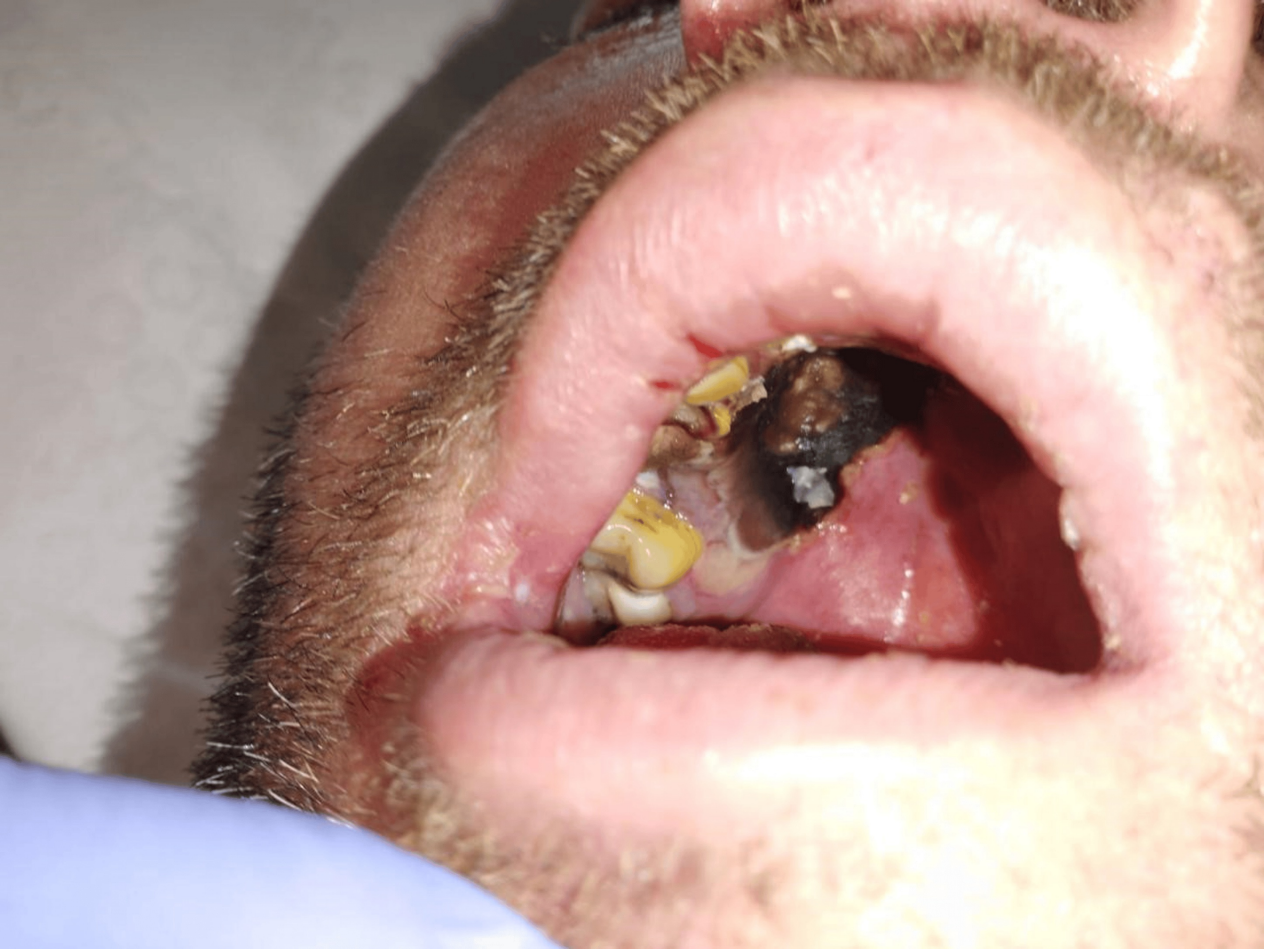
Extraoral photograph showing palatal necrotic lesion

Ten hours after the surgery, the patient developed right-sided ptosis and paresthesia around the maxillary and forehead region. Pharyngoscopy revealed a small necrotic lesion on the right side of the hard palate (Figure 3).

Based on the above signs and symptoms, mucormycosis was suspected as a possible cause, and amphotericin B (400 mg, 0.25 mg/kg IV) was added to the patient’s antibiotic regimen. The patient showed poor response to treatment and his condition deteriorated, developing complete mydriasis of the right pupil, ophthalmoplegia, and loss of vision the following day. Due to the significant amount of necrotic tissue on the hard palate and nasopharynx, a necrotomy was performed by the maxillofacial surgeon on October 17, 2023.

**Figure 4:**
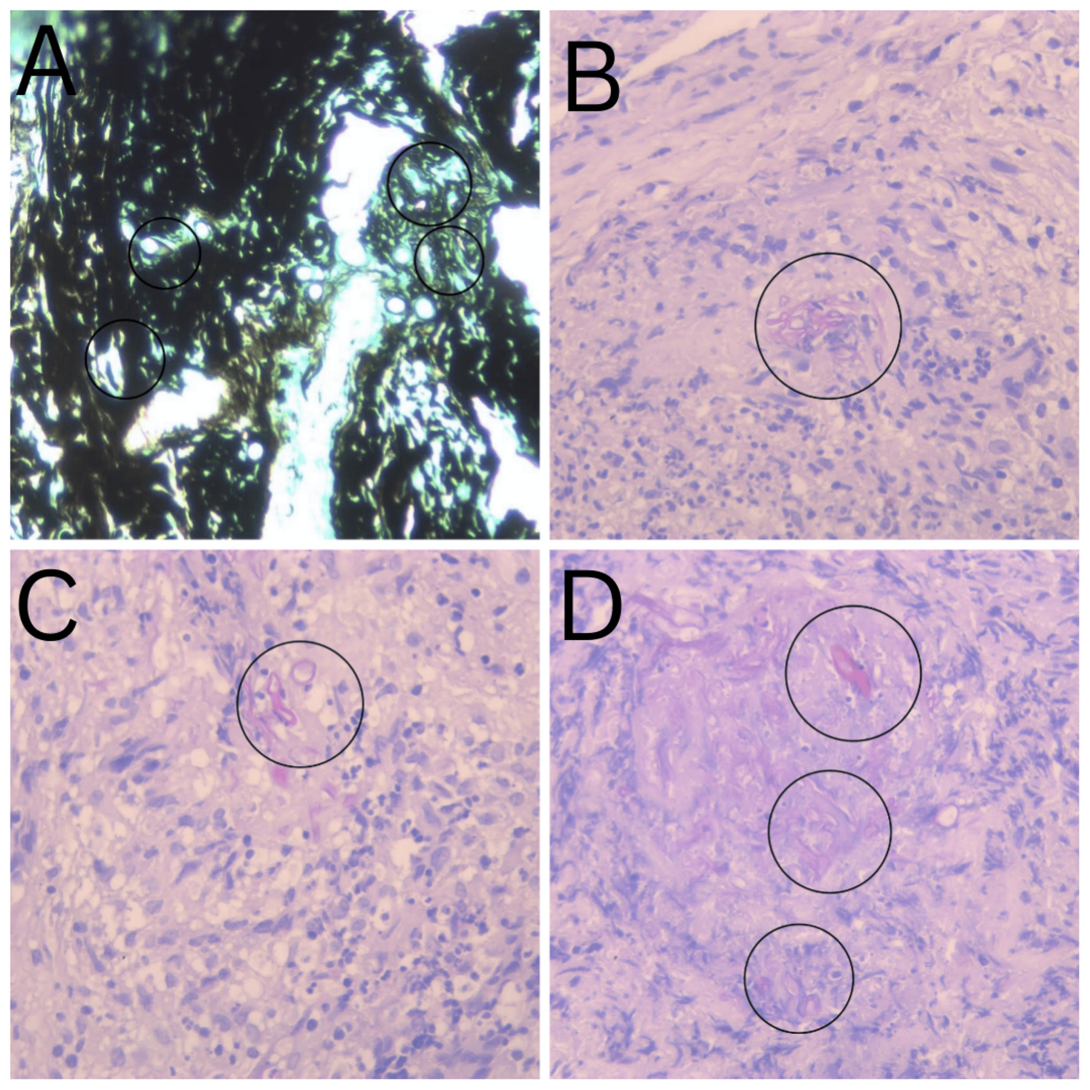
Pathology Hematoxylin and eosin stain (H&E) photomicrographs (B, C, D) show mucormycosis organism fragments with necrotic and inflammatory changes. Grocott–Gömöri methenamine silver stain (GMS) shows the gray and black appearance of mucormycosis organisms with peripheral outline (A).

On October 20, 2023, the histopathology results revealed inflamed and necrotic soft tissues that contained 90-degree branching and non-septating hyphae on hematoxylin and eosin stain (H&E) and Grocott-Gömöri stains, and a final diagnosis of mucormycosis was made (Figure 4). Culture from the necrotic tissue was done and the mold *Rhizopus *was identified by MALDI-TOF MS (Bruker, Germany).

**Figure 5:**
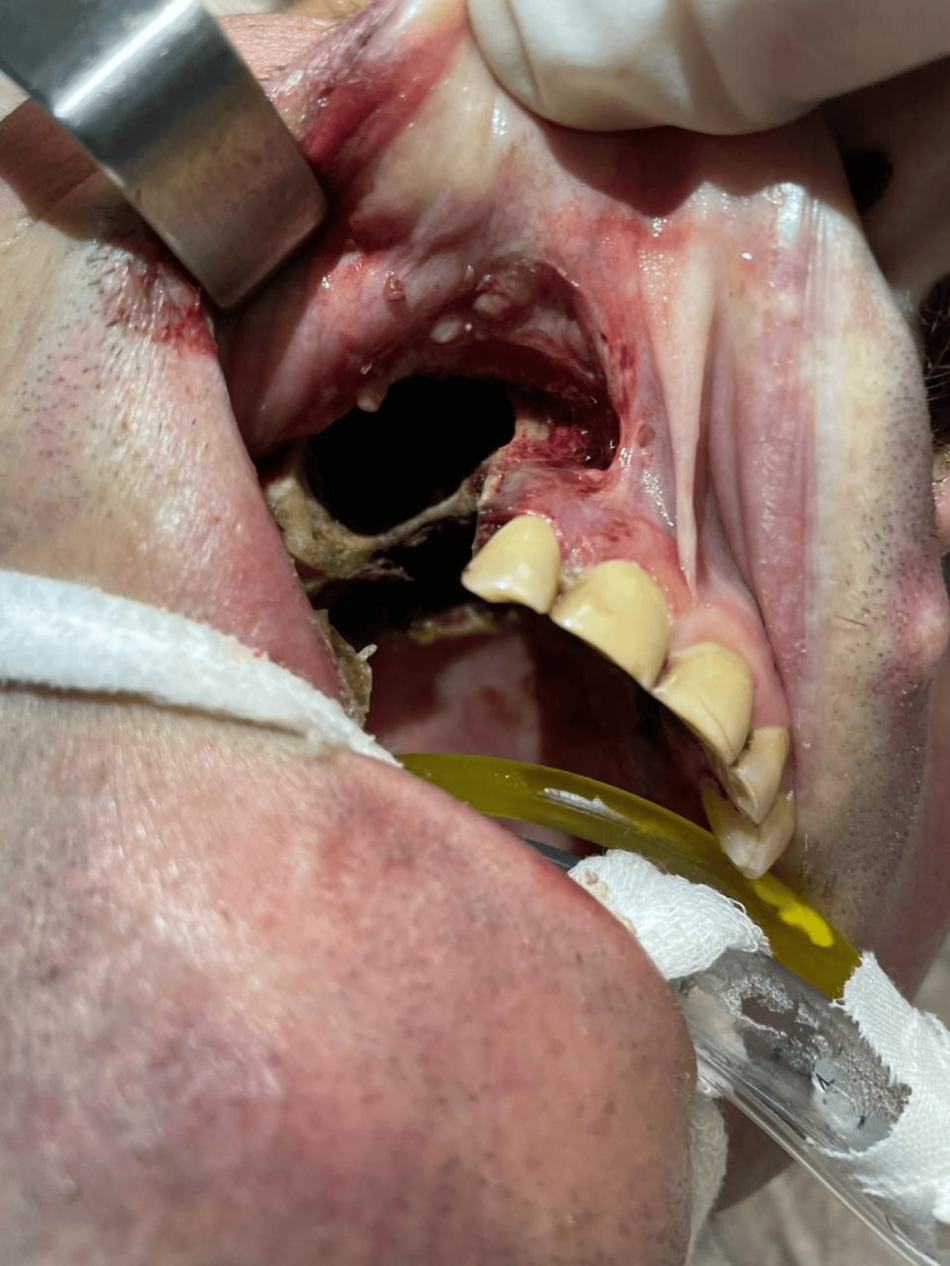
Extraoral photograph after necrotomy and resection of the maxillary bone

The patient showed poor response to the treatment. He underwent repeated maxillary necrotomy with resection of the maxillary bone on October 26, 2023, and remained on mechanical ventilation (photograph after resection of the maxillary bone is shown in Figure 5). As depicted by laboratory values in Table 2, the patient's condition continued to decline, leading to multiorgan failure and death on October 30, 2023.

**Table 2:** Laboratory values on October 26, 2023 CRP: C-reactive protein; INR: international normalized ratio

Parameters	Patient lab values	Normal lab values
Red blood cells	3.5 million/mm3	4.3-5.9 million/mm3
White blood cells	15.74 × 10^9^/L	4.5 × 10^9^/L-11 × 10^9^/L
Platelets	310 × 10^9^/L	150-450 × 10^9^/L
INR	1.7	0.8-1.1
Creatinine	470 µmol/L	59-104 µmol/L
CRP	241 mg/dL	<10 mg/dL
Glucose	221 mg/dL	70-99 mg/dL
Procalcitonin	19.19 ng/ml	<0.1 ng/ml

## Discussion

Mucormycosis is associated with high mortality. Mortality can range from 40% to 80% depending on the underlying conditions affecting the patient [11]. Improved survival is seen with early disease detection and a combination of treatments, while factors related to lower survival rates include delayed diagnosis and treatment, hemiparesis, and renal disease [12]. Physicians should maintain vigilance for potential mucormycosis in high-risk populations and pursue histopathologic testing as soon as possible in suspected cases, which remains the cornerstone of microbiological diagnosis. Physicians should interpret culture findings based on the clinical picture, as contamination with *Mucorales *species is relatively common.

Using advanced diagnostic techniques, including PCR and MALDI-TOF MS, may be a useful adjunct to facilitate an earlier diagnosis in culture-negative cases [2]. Treatment of mucormycosis is a combination of surgery, when possible, correction of underlying risk factors, and aggressive antifungal therapy [11]. Currently, amphotericin B and its lipid formulations remain the standard of care. Early data suggest that posaconazole and isavuconazole may be used as salvage therapy for patients unresponsive to amphotericin B [13,14]. Another study suggested that patients treated with a combination of polyene-caspofungin therapy had superior success and Kaplan-Meyer survival time, compared to patients who received polyene monotherapy [15].

## Conclusions

Mucormycosis is an invasive fungal infection with a grave prognosis. Clinicians should maintain high suspicion in patients with predisposing risk factors. Further attempts should be made to aid in the early diagnosis of the infection and prompt management of the patient. In addition, more high-quality research is needed to develop and standardize new diagnostic methods and alternative antifungal treatment regimens for cases unresponsive to amphotericin B.
